# Co-Design in the Development of a Mobile Health App for the Management of Knee Osteoarthritis by Patients and Physicians: Qualitative Study

**DOI:** 10.2196/17893

**Published:** 2020-07-10

**Authors:** Kelly J Mrklas, Tanya Barber, Denise Campbell-Scherer, Lee A Green, Linda C Li, Nancy Marlett, Jean Miller, Brittany Shewchuk, Sylvia Teare, Tracy Wasylak, Deborah A Marshall

**Affiliations:** 1 Strategic Clinical Networks Provincial Clinical Excellence Alberta Health Services Calgary, AB Canada; 2 Department of Community Health Sciences Cumming School of Medicine University of Calgary Calgary, AB Canada; 3 Enhancing Alberta Primary Care Research Networks Department of Family Medicine University of Alberta Edmonton, AB Canada; 4 Department of Family Medicine University of Alberta Edmonton, AB Canada; 5 Department of Physical Therapy University of British Columbia Vancouver, BC Canada; 6 Arthritis Research Canada Richmond, BC Canada; 7 O'Brien Institute for Public Health University of Calgary Calgary, AB Canada; 8 Community Rehabilitation and Disability Studies, Department of Community Health Sciences Cumming School of Medicine University of Calgary Calgary, AB Canada; 9 Faculty of Nursing University of Calgary Calgary, AB Canada

**Keywords:** health services research, app, knee osteoarthritis, community-based participatory research

## Abstract

**Background:**

Despite a doubling of osteoarthritis-targeted mobile health (mHealth) apps and high user interest and demand for health apps, their impact on patients, patient outcomes, and providers has not met expectations. Most health and medical apps fail to retain users longer than 90 days, and their potential for facilitating disease management, data sharing, and patient-provider communication is untapped. An important, recurrent criticism of app technology development is low user integration design. User integration ensures user needs, desires, functional requirements, and app aesthetics are responsive and reflect target user preferences.

**Objective:**

This study aims to describe the co-design process for developing a knee osteoarthritis minimum viable product (MVP) mHealth app with patients, family physicians, and researchers that facilitates guided, evidence-based self-management and patient-physician communication.

**Methods:**

Our qualitative co-design approach involved focus groups, prioritization activities, and a pre-post quality and satisfaction Kano survey. Study participants included family physicians, patient researchers and patients with knee osteoarthritis (including previous participants of related collaborative research), researchers, key stakeholders, and industry partners. The study setting was an academic health center in Southern Alberta.

**Results:**

Distinct differences exist between what patients, physicians, and researchers perceive are the most important, convenient, desirable, and actionable app functional requirements. Despite differences, study participants agreed that the MVP should be electronic, should track patient symptoms and activities, and include features customized for patient- and physician-identified factors and international guideline-based self-management strategies. Through the research process, participants negotiated consensus on their respective priority functional requirements. The highest priorities were a visual symptom graph, setting goals, exercise planning and daily tracking, and self-management strategies. The structured co-design with patients, physicians, and researchers established multiple collaborative processes, grounded in shared concepts, language, power, rationale, mutual learning, and respect for diversity and differing opinions. These shared team principles fostered an open and inclusive environment that allowed for effective conceptualization, negotiation, and group reflection, aided by the provision of tangible and ongoing support throughout the research process, which encouraged team members to question conventional thinking. Group-, subgroup-, and individual-level data helped the team reveal how and for whom perspectives about individual functional requirements changed or remained stable over the course of the study. This provided valuable insight into how and why consensus emerged, despite the presence of multiple and differing underlying rationales for functional requirement prioritization.

**Conclusions:**

It is feasible to preserve the diversity of perspectives while negotiating a consensus on the core functional requirements of an mHealth prototype app for knee osteoarthritis management. Our study sample was purposely constructed to facilitate high co-design interactivity. This study revealed important differences between the patient, physician, and researcher preferences for functional requirements of an mHealth app that did not preclude the development of consensus.

## Introduction

### Background

Knee osteoarthritis is an inflammatory condition affecting over 25% of middle-aged to older adults [1], causing significant disability and reduced health-related quality of life. Knee osteoarthritis is a significant, growing economic and health burden to afflicted individuals and the society at large [2,3] and is one of the most prevalent diseases worldwide [4].

Fortunately, mobile health (mHealth) technology, defined as “medical and public health practice supported by mobile devices, such as mobile phones, patient monitoring devices, personal digital assistants, and other wireless devices,” including apps [4], holds great promise for advancing the treatment and management of chronic diseases, such as knee osteoarthritis. Mobile technology is the fastest spreading technology in modern history, expanding more than 28-fold, from an average of 3.7 mobile cellular subscriptions per 100 individuals in 1997 to 103 per 100 individuals in 2017 [5]. With thousands of apps released daily [6], mobile technology has been ascribed *limitless potential* to enhance patient and provider access to evidence-based, effective health care resources, at a lower cost [7].

Importantly, recent osteoarthritis treatment and management guidelines endorse patient self-management as a means to increase self-awareness of symptoms and better prepare patients to assume active roles in shared medical decision making [8]. mHealth can enable patient self-management through the use of wearable aides for activity monitoring and behavioral change using personalized, real-time feedback [9], through acquisition of new knowledge, and by enhancing patient-provider communication [10,11].

Despite the doubling of osteoarthritis-targeted mHealth apps over the last decade, both app volume and app research focused on knee osteoarthritis are incongruent with its high global prevalence [12]. Most current apps fail to meet patient and provider expectations for disease management, data sharing, and enhancing communication [8,13,14]. Furthermore, although users demonstrate high interest and demand for health and medical apps [14], the vast majority fail to retain even a third of users after 90 days because of infrequent use, high user turnover, and low loyalty [15].

Unfortunately, research on knee osteoarthritis app development, assessment, and effectiveness [4,8,14] is generally lacking [13], as is a fulsome understanding of the documented or novel barriers that lead to app discontinuity to inform meaningful design improvements. Multiple existing studies document key issues, including functionality, content and content personalization, interactivity, behavior change theory integration, and sustained use [13-17].

Calls to adopt more participatory, coproduction approaches to technology design have emerged over the last decade in many disciplines [18-20]; lack of co-design is a recurring criticism of app development and design. User involvement is crucial to ensure that needs, preferences, functional requirements, and app aesthetics are responsive, reflect, and meet end user needs [18,19]. Empirically tested mHealth apps oriented to and built specifically with and for the needs, preferences, and activities of patients with knee osteoarthritis and their providers [8] are needed.

The call for apps that focus specifically on knee osteoarthritis self-management, patient-clinician decision support, and shared decision making [8] has not yet been answered; the anticipated benefits of mHealth have not been fully realized.

### Objectives

This study aimed to describe the co-design process of knee osteoarthritis minimum viable product (MVP) app development with and for patients and family physicians. An MVP (versus a more comprehensive design product) was chosen because of its specific focus on establishing key end user requirements, meeting early adopter needs, and supporting ongoing iterative research–focused app development, at optimized time and cost [21]. The intent of MVP app development was to facilitate guided self-management, provide evidence-based information to patients and physicians, and facilitate communication while addressing patient needs and challenges [22]. We sought to understand whether and how patient and provider preferences for the functional requirements of a knee osteoarthritis app differ, and whether patient-provider consensus was possible, in the development of an app supporting knee osteoarthritis management [14].

## Methods

### Engaging People With Lived Experience as Research Team Members

This participatory research project engaged people with lived experience of knee osteoarthritis from idea inception through data collection, analysis, and dissemination. People with lived experience provided crucial direction in co-design approach development with team members trained as patient and community engagement researchers (PACERs) [23], informed by the Guidance for Reporting Involvement of Patients and the Public 2.0 checklist [24].

### Research Coalition

Patients, patient researchers, physicians, researchers, decision makers, industry partners, and trainees were engaged to co-design an MVP by combining 3 stakeholder-specific collaborative research approaches. This included a patient-to-patient approach [23] developed by and for patients through PACER in which qualitatively trained patients co-design using a 3-step grounded theory-based research process: (1) *set* to clarify and explore the topic, (2) *collect* to interview, and (3) *reflect* through collaborative analysis. The second approach mobilized tacit health care provider knowledge and was developed by the Enhancing Alberta Primary Care Research Networks (ENACT) team [25]. ENACT supports practice-based research networks and academic and community practitioners conducting research in primary care. The third approach was iteratively developed by team researchers, informed by integrated knowledge translation [26] and collaborative participatory design approach principles [18].

### Conceptual Framework

Conceptual underpinnings are derived from the synthesis-based mHealth design framework for osteoarthritis self-management by Choi et al [8], which highlights evidence-based decision support, osteoarthritis assessment, shared decision making, self-management (such as education, physical activity, feedback, symptom/movement, and joint function monitoring), and data visualization for patients and providers.

### Recruitment

Patient participants, previously recruited through the media, the Arthritis Society, and posters for participation in several preceding PACER studies [22,23,27] were purposively recruited and reinvited to continue their research involvement in this study. Patients were eligible if they reported knee pain “on most days of the month at any time in the past and any pain in the past 12 months” [28]. Family physicians who had either participated or showed interest but were unable to participate in the preceding study [22] were invited by email and asked to nominate a colleague if they could not attend. Physicians were *early majority* [29] practitioners (ie, the first sizable segment of providers to adopt an innovation after seeing others try it) and were recruited to avoid designing around unrepresentative perspectives of *early adopters*. Physicians had a diverse range of experiences in practice settings in Alberta.

Key stakeholders, comprising decision makers from Alberta Health Services, the main provincial health care service provider, and the Alberta Bone and Joint Research Institute, were invited to join the study from inception. Our industry partner was invited to join the third interactive co-design session.

### Study Design

We planned 3 full day, co-design sessions involving semistructured focus groups, ranking and prioritization activities, and a presession and postsession quality and satisfaction (ie, convenience and importance) Kano survey [30]. Sessions were full day, face-to-face meetings hosted at an academic health campus in Calgary, Alberta, Canada (Table 1). Sessions were informed by previous studies [22,23,27,28] and iteratively by findings arising from each co-design session. This study was reviewed and approved by the Conjoint Health Research Ethics Board at the University of Calgary (REB161372).

**Table 1:** Co-design session objectives, methods, and outputs.

Event	Objectives	Methods (analysis)	Outputs
Session 1 (March 27, 2017)	Establish MVP^a^ symptom and quality-of-life measures for patients and physicians 2. Establish parameters for MVP use as communication and self-management tool	Semistructured focus groups (thematic analysis of flip chart data, notes, transcribed notes)	Tool category features: symptoms and activity, red flags/triggers, and guided self-management strategies Summaries, executive summaries
Session 2 (April 18, 2017)	Condense potential functional requirements Determine relative importance and define functionality of MVP requirements Explore how functional requirement use by patients and physicians, to improve patient outcomes	Semistructured focus groups (member check, initial theming and thematic analysis of flip chart data, notes, transcribed notes) Provisional dot voting (frequency counts used as a provisional prioritization criteria for each group)	Categorized tool features Inputs: goal setting, context, symptom tracking, activity tracking, plans/strategies, prognosis prediction (input) Interaction reminders: daily, event-based, periodic outputs; and feedback to patients, physician summary, red flags, prognosis prediction (output) Summaries, executive summaries
Kano presurvey and postsurveys (October 1, 2017 and October 5, 2018)	Determine how stakeholders (patients, physicians, researchers, and decision makers) rated functional requirements by importance and convenience before and after group introduction and review of MVP	Mean (SD) importance score by participant group and all respondents, frequency count by category and participant group) Convenience scores (reported by participant group, frequency count by category and participant group)	Quantified importance/convenience scores for functional requirements Thematic analysis of qualitative comments by group (if required)
Session 3 (October 3, 2018)	To review MVP appearance (wireframes) and mock function, and provide feedback on functional requirements for design iteration with development team and gather a definitive prioritization and ranking of functional requirements for inclusion in the final MVP using dot voting	Semistructured focus group discussion (member check, initial theming and thematic analysis of notes: main take-aways) Dot voting (frequency counts/range on task 1: must-have, won’t-have prioritization and task 2: desirability and actionability prioritization, reported by participant group and all respondents)	Must-have, won’t-have dot voting results by participant group and for all respondents, for each functional requirement Desirability and actionability dot voting results, by participant group, all respondents, for each functional requirement

^a^MVP: minimum viable product.

### Co-Design Sessions 1 to 3

A total of 7 *a priori* objectives defined the problem, needs, and scope and functions of the proposed MVP development (Table 1). Co-design participants were separated into 2 multistakeholder groups (two groups at S1 and S2, only one multistakeholder group at S3), comprising the patients, physicians, researchers, and decision makers, led by research staff and 2 note takers each. This same format and procedures were applied to all sessions.

S1 and S2 guiding questions and activities sought participant perspectives on symptoms and MVP functionality, including how physicians and patients measure and identify red flag symptoms that trigger a family physician visit, how perceived quality of life is affected by different symptoms, patient and provider experience, and observed symptom variation. The team investigated symptom prioritization and rating by both patients and physicians using the Western Ontario and McMaster Universities Arthritis Index (WOMAC) Numerical Rating Scale 3.1 index [31]. The WOMAC is a validated, 24-item, self-administered knee and hip osteoarthritis index that assesses 3 dimensions: pain, disability, and joint stiffness. These data established broad MVP use parameters, including supporting patient-physician communication and guided self-management, exploring MVP benefits/limitations, and potential capability for research-guided self-management.

The findings generated at earlier co-design and dot voting/prioritization activities [32,33] were reconsidered by participants during each session, and they were iteratively used to help identify and define key functional requirements. This iterative process was also used to surface discrepant views for individual and group reflections, and subsequent discussion [34].

Dot voting prioritization involved participants applying 2 each of *must-have* (green) and *won’t-have* (red) stickers to their priority requirements, in any desired configuration. Similarly, S3 participants were given 10 each of desirability (blue) and actionability (yellow) stickers to rank applicable requirements. Votes were tallied by group (patients, physicians, and researchers) and combined with other findings for prioritization. Both sessions helped define the functional requirement characteristics.

In the 6 months preceding S3, our industry partner applied early findings to technical analysis and data consolidation and generated recommendations for appropriate technologies and basic functional requirements. MVP mood boards (visual guide of skeletal framework) were developed for S3 to illustrate page layout, content arrangement, function range and prioritization, display rules, and the effects of different scenarios on app display. Boards were used to collect feedback and generate definitive MVP functional requirement prioritization.

A Kano survey, conceptually grounded in the two-factor motivational theory by Herzberg et al [30,35], assessed satisfaction with proposed functionalities, and the degree to which functionalities were required for satisfaction [36]. The Kano survey is based on the theory of attractive quality, often used to assess and relate customer satisfaction with specific quality attributes [37]. In health care, it is used for function-satisfaction interface [38] assessments, including patient perceptions of service quality and quality expectations, quality elements, patient-provider relationships, satisfaction, and assessing how expectations vary with increased awareness to inform appropriate and aligned service requirement design [38].

A web-enabled Kano survey, comprising 10 three-part question clusters, was emailed to all co-design participants 3 days preceding and immediately following S3. The survey was accompanied by a link to click-through MVP static wireframes for independent review by participants (Multimedia Appendix 1) [36,39].

### Data Collection and Analysis

Table 1 itemizes key data collection events, objectives, methods, and planned outputs. S1 and S2 discussions were audio recorded and key points transcribed by a research assistant, supplemented by findings from the note takers. Text was analyzed line by line, and important patterns pertaining to session objectives and research questions were identified [40]. Action-oriented themes were framed as *take-aways* and refined for coherence [40]. Summaries were generated to inform iterative co-design.

The Kano survey results were collated, frequencies tabulated, and visualized graphically by requirement and stakeholder group (ie, patients, physicians, and researchers). Functional requirements were ranked based on the combined study findings. Mean importance (SD) was calculated for each requirement and for each participant group, and an adjusted mean importance (SD) was calculated across all respondents. Summaries describing and quantifying the co-design process and its outputs were generated for each phase. Survey comments were collated and analyzed for important patterns [40].

Dot voting [32,33] was used to identify, rank, and prioritize functional requirements on convenience dimensions (ie, must-have, won’t-have, desirable, and actionable). Frequencies (range) were presented for each dimension by the participant group (ie, patients, physicians, researchers, and all participants) and summarized.

## Results

### Co-Design Participants

A total of 28 unique co-design participants (13 males and 15 females) took part in at least one session. Overall, 4 patients, 5 physicians, 12 researchers (including 7 team, 3 PACERs, and 2 ENACT researchers), 3 trainees, and 2 decision makers took part in the co-design process to refine concepts, functional requirements, and their relative priority (Table 2). Two industry partner team members observed and interacted at the S3 discussion, and neither completed Kano surveys and dot voting.

**Table 2:** Co-design participant demographics (sessions 1-3).

Session and participants by group			Male, n		Female, n		Participants by session, n
**Session 1**							
	Patients	2		2		4	
	Physicians	1		1		2	
	UCalgary^a^/academic researchers	2		4		6	
	ENACT^b^ researchers	1		1		2	
	PACER^c^	0		3		3	
	Decision makers	1		1		2	
	Trainees	0		0		0	
Total Session 1			7		12		19
**Session 2**							
	Patient	2		0		2	
	Physicians	0		2		2	
	UCalgary/academic researchers	2		4		6	
	ENACT researchers	1		1		2	
	PACER	0		2		2	
	Decision makers	1		1		2	
	Trainees^d^	3		0		3	
Total Session 2			9		10		19
**Session 3**							
	Patients	2		2		4	
	Physicians	1		1		2	
	UCalgary/academic researchers	1		3		4	
	ENACT researchers	1		1		2	
	PACER	0		3		3	
	Decision makers	0		0		0	
	Trainees	0		0		0	
	Industry partners	2		0		2	
Total Session 3			7		10		17

^a^UCalgary: University of Calgary.

^b^ENACT: Enhancing Alberta Primary Care Research Networks team.

^c^PACER: patient and community engagement researchers.

^d^Computer Science trainees, University of Calgary.

### Co-Design Sessions 1 and 2: Focus Group Findings

S1 discussions generated a unanimous agreement on basic MVP features and purpose, scope, patient/physician preferences and rationale, design action items, and evolving refinements of app functionalities (Textbox 1). Overall, the group thought that the MVP should have the following:

- Be electronic (ie, cell phone or web-enabled).
- Help patients manage symptoms and activity.
- Include customized red flags/triggers.
- Include evidence-based guided self-management strategies (eg, output structured by the Osteoarthritis Research Society International guidelines), including pain, weight management and aids, scheduling for self-management strategies, track progression, and report history (eg, activity, symptoms, red flags), and self-management plan for physician visits [31,41].

**Sessions 1 and 2 summary: participant-selected inputs, outputs and interactions/reminders.**

Goal setting, context (input)

- Patient-customized goals defined on first app use (symptoms, quality of life, activity and linked to custom activities, reminders). Tracking of activities as output. Patient customizable comorbidities; visualized (homunculus), symptoms, activity history, previous plans, strategies

Symptom tracking (input)

- Dimensions: pain, stiffness, function, others (swelling, warmth, and inflammation) by validated, evidence-based tools. Patient-guided entry: threshold approach (eg, visual analog scale 0-10 provided, if higher than predetermined threshold, prompts location, duration, and intensity). Journal for situation-specific symptom record. Symptom history as output

Activity tracking (input)

- Patient-customized goal setting and exercises (evidence-based physiotherapy exercises). Exercise resource links per patients’ needs

Plans, strategies (input)

- Sliders: My Exercise Plan, My Diet, My Medication (prescription, topical, and over the counter), and My Assistive Devices/Supports (aids, accessories, and other therapies). Categories to include attempted therapies with evidence-based *drop down* list and customizability. Ranking function to gather customized patient data about utility of strategies

Prognosis, prediction (input)

- Algorithm to capture patient symptoms, other information, and prediction of osteoarthritis severity. Patients prompted to enter information, context, and demographic information on first app use with customizable symptom tracking (time and frequency). Prognosis prediction as output, graphic visualization for patients/providers. Minimum input, thresholds required.

Feedback to patients (output)

- Report historical summary of goals, symptoms, activity, plans/strategies on patient dashboard. Provider summary graph as a separate dashboard with one chart containing symptoms and activity.

Red flags (output)

- Automated, predefined rules to capture symptoms, activity. Data considered red flags or signs stimulate action (eg, physician visit/emergency department). Patient button to journal red flags as events to share with providers

Prognosis, prediction [42] (output)

- On the basis of symptom inputted by patient, graph displays symptom fluctuation; sharable with providers

Daily, event-based, and periodic (interactions and reminders)

- Customized input for daily, event-based, periodic reminders. Reminders seen as small red sign with numbers. Custom daily input used for symptom/activity tracking. Periodic input (other than initial patient input) for goal setting, plans, strategies. Automated reminders aligned with exercises, goals, pain, etc

S1 discussions generated additional insights considered during S2 dot voting/prioritization activities. To facilitate further refinement and discussion, participants decided to use S2 dot voting to help generate consensus during their discussions rather than dot voting to prioritize as initially planned (Textbox 1).

When discrepancies surfaced, these were highlighted during and at the end of agenda sections at each session, and then revisited for further exploration during report back times and group discussions [34]. Participants eventually agreed to and provided details on 12 functional requirements (*inputs* [n=5]: goal setting and context, symptom tracking, activity tracking, plans or strategies, and prognosis prediction; *interaction reminders* [n=3]: daily, event-based and periodic reminders; and *outputs* [n=4]: feedback to patients, physician summary, red flags, and prognosis prediction). Participants advocated for validated instruments and high-quality evidence sources for medically relevant requirements identified by researchers. S1 and S2 findings were consolidated and requirements refined with the industry partner to inform S3 findings.

### Session 3 Findings

Overall, 17 individuals (7 males and 10 females) attended session 3, including 4 patients and 4 providers, 2 physicians and 2 ENACT researchers, 3 PACERs, and 2 industry partner facilitators. S3 was preceded and followed by Kano survey assessments and involved in-depth consideration of the refined functionalities that emerged from S2. Cumulative findings were reviewed and checked by participants at the start and high-priority requirements refined iteratively through S3 assessments, prioritization activities, and group discussions.

### Kano Survey: Importance

The Kano surveys helped qualitatively describe, enumerate, and reveal evolving co-design processes and outputs (Tables 3 and 4).

Importance assessments revealed the *top 6* functional requirements: track pain symptoms, visual graph of symptoms, self-management strategies, setting goals and follow through, track functional impairment, and plan exercises and daily tracking. Although differences in perceived importance arose between participant groups, the *top 6* requirements remained the same pre- and post-Kano survey, summarized in Table 5.

Patients scored self-management as highly important and patients referred to the MVP as a source of motivation, control, planning, and a means of encouraging positive behavior. Patients appreciated having knee osteoarthritis management functions in a single spot, using the app to facilitate a physician-patient interaction, and customizing and tracking progress over time.

**Table 3:** Presession Kano survey: importance by participant group (n=13).

App feature	Importance (9-point Likert scale: 1=not at all important to 9=extremely important)									Mean adjusted importance (n=13)		
	Patients (n=6)		Physicians (n=3)			Researchers (n=3)						
	Mean (SD)	Rank^a^		Mean (SD)	Rank^a^		Mean (SD)	Rank^a^			Mean (SD)	*Overall rank* ^a,b^
If the app could show you a graph of your 7-year osteoarthritis severity prediction, how do you feel?	7.3 (1.03)	6		4.3 (2.08)	8		6.0^c^ (1.41)	9			6.27 (1.85)	*9*
If the app could help you to set goals and follow through, how do you feel?	8.2 (0.98)	2		8.3 (0.58)	2		8.7 (0.58)	1			8.33 (0.78)	*1*
If the app could help you set a plan with various exercises and track them daily, how do you feel?	6.7 (2.94)	7		8.7 (0.58)	1		7.7 (1.53)	5			7.42 (2.27)	*6*
If the app could allow you to track your pain symptoms over time, how do you feel?	7.8 (1.33)	4		7.7 (1.53)	4		8.3 (0.58)	4			7.92 (1.16)	*4*
If the app could allow you to track your stiffness symptoms over time, how do you feel?	7.5 (1.22)	5		6.0 (2.65)	7		6.3 (1.15)	8			6.83 (1.64)	*7*
If the app could allow you to track your functional impairment symptoms over time, how do you feel?	8.0 (1.26)	3		8.3 (1.15)	2		8.7 (0.58)	2			8.25 (1.06)	*2*
If the app could show you a graph of your symptoms over time, how do you feel?	8.5 (0.84)	1		6.7 (2.08)	6		8.3 (0.58)	3			8.00 (1.35)	*3*
If the app could give you strategies to help you self-manage your arthritis, how do you feel?	7.3 (1.21)	6		8.0 (0)	3		7.3 (1.15)	7			7.50 (1.00)	*5*
If the app could let you flag certain days where arthritis impacted your plans, how do you feel?	5.2 (2.56)	9		4.0 (2.0)	9		7.7 (0.58)	6			5.50 (2.39)	*10*
If the app could give you reminders to update your information (symptoms, exercise, goal tracking), how do you feel?	6.3 (2.16)	8		7.0 (1.0)	5		5.7 (0.58)	10			6.33 (1.61)	*8*

^a^Rank subjectively assessed based on a combination of mean scores and overall mean adjusted scores, 1=highest rank, 10=lowest rank.

^b^Italics emphasize the overall rank for each functional requirement.

^c^2 responses only.

**Table 4:** Postsession Kano survey: importance by participant group (n=12).

App feature	Importance (9-point Likert scale 1=not at all important to 9=extremely important)						Mean adjusted importance (n=12)		
	Patients (n=7)		Physicians (n=2)		Researchers (n=3)				
	Mean (SD)	Rank^a^	Mean (SD)	Rank^a^	Mean (SD)	Rank^a^		Mean (SD)	*Overall rank* ^a,b^
If the app could show you a graph of your 7-year osteoarthritis severity prediction, how do you feel?	5.90 (2.19)	7	5.50 (2.12)	8	6.70 (1.15)	6		6.00 (1.86)	*8*
If the app could help you to set goals and follow through, how do you feel?	7.00 (2.52)	4	9.00 (0)	1	6.00 (1.73)	7		7.10 (2.23)	*4*
If the app could help you set a plan with various exercises and track them daily, how do you feel?	6.60 (1.81)	5	8.50 (0.71)	3	6.00 (2.83)	8		6.80 (1.89)	*6*
If the app could allow you to track your pain symptoms over time, how do you feel?	7.40 (1.27)	2	7.50 (0.71)	6	8.30 (0.58)	3		7.70 (1.07)	*1*
If the app could allow you to track your stiffness symptoms over time, how do you feel?	5.60 (2.23)	9	3.00 (1.41)	9	8.00 (1.00)	4		5.80 (2.42)	*9*
If the app could allow you to track your functional impairment symptoms over time, how do you feel?	5.90 (2.34)	8	8.50 (0.71)	2	8.30 (0.58)	2		6.90 (2.19)	*5*
If the app could show you a graph of your symptoms over time, how do you feel?	7.00 (1.83)	3	7.50 (2.12)	4	8.70 (0.58)	1		7.50 (1.68)	*2*
If the app could give you strategies to help you self-manage your arthritis, how do you feel?	7.70 (1.11)	1	7.50 (2.12)	5	7.00 (1.00)	5		7.50 (1.17)	*3*
If the app could let you flag certain days where arthritis impacted your plans, how do you feel?	5.10 (1.77)	10	2.50 (0.71)	10	5.70 (4.16)	10		4.80 (2.48)	*10*
If the app could give you reminders to update your information (symptoms, exercise, goal tracking), how do you feel?	6.30 (1.89)	6	6.00 (2.83)	7	6.00 (2.00)	9		6.20 (1.85)	*7*

^a^Subjectively assessed rank based on mean scores and overall mean adjusted scores, 1=highest rank, 10=lowest rank.

^b^Italics emphasize the overall rank for each functional requirement.

**Table 5:** Functional requirement importance ranking: presession and postsession 3 Kano survey.

Presession Kano survey results	Rank^a^	Postsession Kano survey results	Rank^a^
Set goals and follow through	1	Track pain symptoms	1
Track functional impairment symptoms	2	Visual graph of symptoms	2
Visual graph of symptoms	3	Self-management strategies	3
Track pain symptoms	4	Set goals and follow through	4
Self-management strategies	5	Track functional impairment symptoms	5
Plan exercises and daily tracking	6	Plan exercises and daily tracking	6
Track stiffness symptoms	7	Reminders to update info	7
Reminders to update info	8	7-year osteoarthritis severity prediction	8
7-year osteoarthritis severity prediction	9	Track stiffness symptoms	9
Flag days	10	Flag days	10

^a^1=highest rank, 10=lowest rank.

### Kano Survey: Convenience

There were clear alignments and differences in how patients, physicians, and researchers assessed the convenience of MVP requirements (Multimedia Appendix 2).

Patients’ presession *must-have* features overlapped with that of the physicians (eg, planning exercises and daily tracking) and expanded in number postsurvey. Setting a plan with exercises and daily tracking remained *must-haves* for patients throughout. Patients scored a 7-year osteoarthritis severity prediction as attractive presession and postsession; researchers and physicians were indifferent. Researchers reported few *must-haves* and aligned with physicians (ie, tracking functional impairment, graphing symptoms, and the ability to plan exercises and track daily).

The *top 6* functional requirements generated by convenience assessments differed from importance assessments in only 2 ways: the inclusion of reminders and the shifting of self-management strategies to a slightly lower rank. Stiffness symptom tracking and flags remained the lowest.

On the basis of the cumulative S1 to S3 findings and discussions, requirements were relabeled into 7 categories, as shown in Table 6.

**Table 6:** Session 3 dot voting results: revised functional requirement categories and summary of must-have and won’t-have features.

Functional requirements	Must-have features				Won’t-have features			
	Patients, n	Physicians, n	Researchers^a^, n	Total, n	Patients, n	Physicians, n	Researchers^a^, n	Total, n
Symptoms graph and summary (charts, diagrams to visualize symptoms, goal achievement, context, and communication)	4	1	3	8	0	0	0	0
Severity prediction (7-year osteoarthritis severity prediction tool [42], WOMAC^b^)	0	0	0	0	3	1	0	4
Setting goals (shared goal setting including work, chores, sports, and hobbies)	0	1	2	3	1	0	0	1
Tracking activity (for events and outcomes, including activities, pain swelling, function, mood, fatigue and interventions, plans, and activities)	0	0	0	0	0	0	0	0
Reminders (reminders to update customized patient information)	0	0	0	0	0	0	3	3
Flags (flags for identifying arthritis burdensome days)	0	0	0	0	2	1	2	5
Information and strategies (self-management strategies including exercises, other conditions, medications, red flags, local resources)	2	0	0	2	0	0	0	0

^a^Missing data (n=1).

^b^WOMAC: Western Ontario and McMaster Universities Arthritis Index.

### Session 3 Dot Voting Findings: Must-Have, Won’t-Have, Desirability, Actionability

There was a strong preference for a symptom graph and summary (Table 6), consistent with previous assessments. Although physicians and researchers rated setting goals as a *must-have* feature, patients favored information and strategies.

Flags was perceived as the highest *won’t-have* requirement (n=5). Patients (n=3) and physicians (n=1) *did not want* severity prediction, which was consistent with physician importance, but contrary to patient importance findings (Tables 3 and 4). Researchers did not want reminders (n=3).

The most *desirable* MVP features were: symptoms graph and summary, setting goals, information and strategies, severity prediction, tracking activity, and reminders, with preference variability for severity prediction and reminders, consistent with importance and convenience assessments (Multimedia Appendix 3). The least *desirable* requirement was *flags*, which was unchanged from other assessment findings.

*Actionable* requirements by descending frequency were symptoms graph and summary, tracking activity, setting goals, severity prediction, information and strategies, reminders, and flags (Multimedia Appendix 4). With the exception of severity prediction, the findings were highly consistent.

## Discussion

### Principal Findings

Co-design participants considered and prioritized MVP functional requirements using iterative, qualitative co-design [26] methods over 3 interactive sessions. Overall, the highest priority requirements included the following: (1) symptoms graph and summary; (2) setting goals; (3) tracking activity; and (4) information and strategies.

Clear differences in preferences existed between stakeholders and were documented throughout the research process. These findings are consistent with other studies examining the priorities and needs of patients and physicians in the management of knee osteoarthritis [22], yet diverse perspectives among patients and physicians did not preclude consensus on MVP functional requirements.

The continuous involvement of participants from inception ensured that the understanding, language, and goals were negotiated. We supported diversity of thought with shared governance and decision making, role clarity, and by maintaining a safe, respectful, transparent research environment.

### Limitations

The pattern of our findings and their consistency with related literature suggest that we captured important alignments and differences between participants through co-design; however, further study to reveal co-design dynamics is required.

It is possible that the patient participants in this study are not representative of *typical* patients with knee osteoarthritis. Individuals actively participating in research over longer periods are more likely to be motivated and have different needs and priorities than those who are less engaged in their own self-management [43-45]. Further validation of these findings within a broader knee osteoarthritis patient population is necessary.

This study was limited in scope to MVP development and purposely involved a smaller user group; however, the research was nested within a larger collaborative research program. Study results were immediately integrated into subsequent co-design and *alpha* testing with a representative sample of patients and physicians (personal communication by DA Marshall, November 2019).

### Comparison With Prior Work

Early S1 and S2 findings revealed high-level agreement on functional requirements among co-design partners. These findings align well with findings from mixed group co-design research by Revenas et al [16,46,47]. However, from the point of initial agreement forward, we observed different, evolving thought patterns from patient, physician, and researcher perspectives. Fluctuating findings reflected emergent mutual understanding and thought diversity [22] among co-design partners. For example, group-level findings varied as a shared understanding of severity prediction emerged, relative to other functional requirements. The discussion revealed a newly developed understanding that the extent and timing of future physical knee osteoarthritis decline was not valued by patients relative to their proximal, preventative needs. Although severity prediction was initially important for patients and physicians, and patients thought it desirable/actionable, it became a clear *won’t-have* feature for patients, and rated less important and convenient for both groups, by the end of S3.

Persistent thought diversity observed throughout our study contrasts with reports of thought homogeneity [16,17,46,47], or what Revenas et al describe as *participant convergence*. *Participant convergence* is the blending of participant perspectives [16,17,46,47], resulting in the inability to discern the diversity of patient and provider voices in the process [17]. As Pilemalm and Timpka report [26], the application of a rigorous co-design approach helped us establish a transparent, safe, and supportive environment for participants to freely express and consider diverse viewpoints [34], and helped preserve thought diversity. Voices were integrated by iteratively raising, airing, and reconciling conflicting interests [34]. These findings also align with a case study by Craven et al [48], in that all participants shared responsibility for identifying discrepancies and contributed to their exploration and reconciliation. In summary, we avoided loss of voice by involving multiple representative co-design partners (ie, patient, physician, and researcher) at each session, using reflective, responsive group processes, supporting open, transparent dialogue and power sharing, developing common language, and actively fostering a culture of mutual respect for the differing, yet equally valued contextual expertise of participants [49,50].

Our findings were highly consistent with key requirements identified by mixed users co-designing rheumatoid arthritis self-management tools, including customization, self-regulation, and exercise planning or follow-up [47]. They were also consistent with general design features of effective electronic health interventions, including social context and support, contacts with intervention, tailoring, and self-management [12,13,51,52].

The continued involvement of participants through each research phase was key [22]. In doing so, we may have avoided common difficulties such as establishing a shared starting point, rationale, and purpose. These and other commonly documented design process challenges (eg, maligned goals and tasks or difficulties turning ideas into concrete app features) [48] did not arise.

From inception, the team discussed expectations for participation and engaged in negotiation and clarification of roles [34]. Shared governance and decision-making principles were operationalized, as evidenced by the groups’ spontaneous repurposing of S2 dot voting methods. Participants openly expressed reservations and negotiated this modification with ease. It is possible we avoided commonly documented partnership challenges [17,48,52] by adopting structural and process components, ensuring adequate resources, and time, by actively engaging our stakeholders in revealing and reconciling multiple, diverging perspectives [34], and by matching participants with research phases [26,53].

The findings are promising; however, systematic assessment and quantification of co-design processes, outcomes, and impact is needed to validate these findings and reveal co-design mechanisms [54].

Finally, there are mixed effectiveness findings and documented challenges associated with mHealth development to support and manage patients with chronic conditions [13,51] such as knee osteoarthritis. These include a lack of sustained app use, diminished product relevance, low daily patient routinization [48,55], high turnover, low app use, and disloyalty [14]. These challenges, coupled with the low reported likelihood of successful app development (an estimated 1 in 10,000 in 2018) [56], necessitate the use of deliberate strategies to optimize interactivity and app relevance for target users.

Our prioritized functional requirements address documented gaps [8], effective design features [12,52], and core mHealth characteristics [57]. For patients, prioritized requirements addressed the inability of patients to track/assess symptoms (eg, pain), a lack of apps to support shared decision making with providers and support more informed patient self-management, and broadened focus beyond education [12,13,51]. For providers, prioritized requirements addressed the ability of an app to facilitate joint function measurement and enhanced decision support.

By reviewing the high-priority, midrange, and low-priority MVP functional requirements, we co-designed an MVP that addresses important, documented barriers for patients and providers in their use of mHealth [51] to manage knee osteoarthritis. The research was carried out in a way that was inclusive of diverse perspectives, yet facilitated consensus.

### Conclusions

In conclusion, this research represents an important intermediate step in an interactive, ongoing dialogue with knee osteoarthritis patients, their providers, and the health research community about mHealth use to support knee osteoarthritis management [22,23,58]. This study offers other researchers tangible rationale for and an example of tailored co-design. The findings reveal how structural and process aspects can facilitate the presence and authenticity of patient, provider, and researcher voices while optimizing an MVP for future research phases.

## Appendix

Multimedia Appendix 1 Session 3 co-design participant surveys.

Multimedia Appendix 2 Kano survey findings: summary of convenience response frequencies pre- and post-session 3, by functional requirement.

Multimedia Appendix 3 Session 3 dot voting results: desirability for each functional requirement by participant type.

Multimedia Appendix 4 Session 3 dot voting results: actionability for each functional requirement by participant type.

## Abbreviations

ENACT: Enhancing Alberta Primary Care Research Networks
mHealth: mobile health
MVP: minimum viable product
PACER: patient and community engagement research
WOMAC: Western Ontario and McMaster Universities Arthritis Index
