# Supplementary material for: Co-Design in the Development of a Mobile Health App for the Management of Knee Osteoarthritis by Patients and Physicians: Qualitative Study
Source: JMIR Mhealth Uhealth. 2020 Jul 10;8(7):e17893. doi: 10.2196/17893 (PMC7382016; doi:10.2196/17893)
Supplement: Multimedia Appendix 1 [file mhealth_v8i7e17893_app1.pdf]

KOASK - Co-design participant survey

This survey helps us understand how useful each potential feature in the KOASK app will be to you.

1. **I am participating in the co-design session as a:**

Mark only one oval.

- Patient
- Physician
- Researcher

1)

2. **If the app could show you a graph of your 7-year osteoarthritis severity prediction, how do you feel?**

Mark only one oval.

- This would be very helpful to me
- This is a basic requirement for me
- This would not affect me
- This would be a minor inconvenience
- This would be a major problem for me

3. **If the app COULD NOT show you a graph of your 7-year osteoarthritis severity prediction, how do you feel?**

Mark only one oval.

- This would be very helpful to me
- This is a basic requirement for me
- This would not affect me
- This would be a minor inconvenience
- This would be a major problem for me

4. **How important is it for the app to show you a graph of your 7-year osteoarthritis severity prediction?**

Mark only one oval.

	1	2	3	4	5	6	7	8	9	
Not at all important										Extremely important

5. **Comments**

2)

6. **If the app could help you to set goals and follow through, how do you feel?**
Mark only one oval.

- This would be very helpful to me
- This is a basic requirement for me
- This would not affect me
- This would be a minor inconvenience
- This would be a major problem for me

7. **If the app COULD NOT help you to set goals and follow through, how do you feel?**
Mark only one oval.

- This would be very helpful to me
- This is a basic requirement for me
- This would not affect me
- This would be a minor inconvenience
- This would be a major problem for me

8. **How important is it for the app to help you to set goals and follow through?**
Mark only one oval.

	1	2	3	4	5	6	7	8	9	
Not at all important										Extremely important

9. **Comments**

3)

10. **If the app could help you set a plan with various exercises and track them daily, how do you feel?**

Mark only one oval.

- This would be very helpful to me
- This is a basic requirement for me
- This would not affect me
- This would be a minor inconvenience
- This would be a major problem for me

11. **If the app COULD NOT help you set a plan with various exercises and track them daily, how do you feel?**

Mark only one oval.

- This would be very helpful to me
- This is a basic requirement for me
- This would not affect me
- This would be a minor inconvenience
- This would be a major problem for me

12. **How important is it for the app to help you set a plan with various exercises and track them daily?**

Mark only one oval.

	1	2	3	4	5	6	7	8	9	
Not at all important										Extremely important

13. **Comments**

4)

14. **If the app could allow you to track your pain symptoms over time, how do you feel?**

Mark only one oval.

- This would be very helpful to me
- This is a basic requirement for me
- This would not affect me
- This would be a minor inconvenience
- This would be a major problem for me

15. **If the app COULD NOT allow you to track your pain symptoms over time, how do you feel?**

Mark only one oval.

- This would be very helpful to me
- This is a basic requirement for me
- This would not affect me
- This would be a minor inconvenience
- This would be a major problem for me

16. **How important is it for the app to allow you to track your pain symptoms over time?**

Mark only one oval.

	1	2	3	4	5	6	7	8	9	
Not at all important										Extremely important

17. **Comments**

5)

18. **If the app could allow you to track your stiffness symptoms over time, how do you feel?**

Mark only one oval.

- This would be very helpful to me
- This is a basic requirement for me
- This would not affect me
- This would be a minor inconvenience
- This would be a major problem for me

19. **If the app COULD NOT allow you to track your stiffness symptoms over time, how do you feel?**

Mark only one oval.

- This would be very helpful to me
- This is a basic requirement for me
- This would not affect me
- This would be a minor inconvenience
- This would be a major problem for me

20. **How important is it for the app to allow you to track your stiffness symptoms over time?**

Mark only one oval.

	1	2	3	4	5	6	7	8	9	
Not at all important	⬭	⬭	⬭	⬭	⬭	⬭	⬭	⬭	⬭	Extremely important

21. **Comments**

6)

22. **If the app could allow you to track your functional impairment symptoms over time, how do you feel?**

Mark only one oval.

- ⬭ This would be very helpful to me
- ⬭ This is a basic requirement for me
- ⬭ This would not affect me
- ⬭ This would be a minor inconvenience
- ⬭ This would be a major problem for me

23. **If the app COULD NOT allow you to track your functional impairment symptoms over time, how do you feel?**

Mark only one oval.

- ⬭ This would be very helpful to me
- ⬭ This is a basic requirement for me
- ⬭ This would not affect me
- ⬭ This would be a minor inconvenience
- ⬭ This would be a major problem for me

24. **How important is it for the app to allow you to track your functional impairment symptoms over time?**

Mark only one oval.

	1	2	3	4	5	6	7	8	9	
Not at all important	⬭	⬭	⬭	⬭	⬭	⬭	⬭	⬭	⬭	Extremely important

25. **Comments**

7)

26. **If the app could show you a graph of your symptoms over time, how do you feel?**
Mark only one oval.

- This would be very helpful to me
- This is a basic requirement for me
- This would not affect me
- This would be a minor inconvenience
- This would be a major problem for me

27. **If the app COULD NOT show you a graph of your symptoms over time, how do you feel?**
Mark only one oval.

- This would be very helpful to me
- This is a basic requirement for me
- This would not affect me
- This would be a minor inconvenience
- This would be a major problem for me

28. **How important is it for the app to show you a graph of your symptoms over time?**
Mark only one oval.

	1	2	3	4	5	6	7	8	9	
Not at all important										Extremely important

29. **Comments**

8)

30. **If the app could give you strategies to help you self-manage your arthritis, how do you feel?**

Mark only one oval.

- This would be very helpful to me
- This is a basic requirement for me
- This would not affect me
- This would be a minor inconvenience
- This would be a major problem for me

31. **If the app COULD NOT give you strategies to help you self-manage your arthritis, how do you feel?**

Mark only one oval.

- This would be very helpful to me
- This is a basic requirement for me
- This would not affect me
- This would be a minor inconvenience
- This would be a major problem for me

32. **How important is it for the app to give you strategies to help you self-manage your arthritis?**

Mark only one oval.

	1	2	3	4	5	6	7	8	9	
Not at all important										Extremely important

33. **Comments**

9)

34. **If the app could let you 'flag' certain days where arthritis impacted your plans, how do you feel?**

Mark only one oval.

- This would be very helpful to me
- This is a basic requirement for me
- This would not affect me
- This would be a minor inconvenience
- This would be a major problem for me

35. **If the app COULD NOT let you 'flag' certain days where arthritis impacted your plans, how do you feel?**

Mark only one oval.

- This would be very helpful to me
- This is a basic requirement for me
- This would not affect me
- This would be a minor inconvenience
- This would be a major problem for me

36. **How important is it for the app to let you 'flag' certain days where arthritis impacted your plans?**

Mark only one oval.

	1	2	3	4	5	6	7	8	9	
Not at all important										Extremely important

37. **Comments**

10)

38. **If the app could give you reminders to update your information (symptoms, exercise, goal tracking), how do you feel?**

Mark only one oval.

- This would be very helpful to me
- This is a basic requirement for me
- This would not affect me
- This would be a minor inconvenience
- This would be a major problem for me

39. **If the app COULD NOT give you reminders to update your information (symptoms, exercise, goal tracking), how do you feel?**

Mark only one oval.

- This would be very helpful to me
- This is a basic requirement for me
- This would not affect me
- This would be a minor inconvenience
- This would be a major problem for me

40. **How important is it for the app to give you reminders to update your information (symptoms, exercise, goal tracking)?**

Mark only one oval.

	1	2	3	4	5	6	7	8	9	
Not at all important										Extremely important

41. **Comments**

Powered by
Google Forms

KOASK - Co-design participant survey (post-session)

This survey helps us understand how useful each potential feature in the KOASK app will be to you.

1. **I am participating in the co-design session as a:**

   Mark only one oval.

   - Patient
   - Physician
   - Researcher

1)

2. **If the app could show you a graph of your 7-year osteoarthritis severity prediction, how do you feel?**

   Mark only one oval.

   - This would be very helpful to me
   - This is a basic requirement for me
   - This would not affect me
   - This would be a minor inconvenience
   - This would be a major problem for me

3. **If the app COULD NOT show you a graph of your 7-year osteoarthritis severity prediction, how do you feel?**

   Mark only one oval.

   - This would be very helpful to me
   - This is a basic requirement for me
   - This would not affect me
   - This would be a minor inconvenience
   - This would be a major problem for me

4. **How important is it for the app to show you a graph of your 7-year osteoarthritis severity prediction?**

   Mark only one oval.

	1	2	3	4	5	6	7	8	9	
Not at all important										Extremely important

5. **Comments**

____
____
____
____
____

2)

6. **If the app could help you to set goals and follow through, how do you feel?**
Mark only one oval.

- ◯ This would be very helpful to me
- ◯ This is a basic requirement for me
- ◯ This would not affect me
- ◯ This would be a minor inconvenience
- ◯ This would be a major problem for me

7. **If the app COULD NOT help you to set goals and follow through, how do you feel?**
Mark only one oval.

- ◯ This would be very helpful to me
- ◯ This is a basic requirement for me
- ◯ This would not affect me
- ◯ This would be a minor inconvenience
- ◯ This would be a major problem for me

8. **How important is it for the app to help you to set goals and follow through?**
Mark only one oval.

	1	2	3	4	5	6	7	8	9	
Not at all important	◯	◯	◯	◯	◯	◯	◯	◯	◯	Extremely important

9. **Comments**

____
____
____
____
____

3)

10. **If the app could help you set a plan with various exercises and track them daily, how do you feel?**

Mark only one oval.

- This would be very helpful to me
- This is a basic requirement for me
- This would not affect me
- This would be a minor inconvenience
- This would be a major problem for me

11. **If the app COULD NOT help you set a plan with various exercises and track them daily, how do you feel?**

Mark only one oval.

- This would be very helpful to me
- This is a basic requirement for me
- This would not affect me
- This would be a minor inconvenience
- This would be a major problem for me

12. **How important is it for the app to help you set a plan with various exercises and track them daily?**

Mark only one oval.

	1	2	3	4	5	6	7	8	9	
Not at all important										Extremely important

13. **Comments**

4)

14. **If the app could allow you to track your pain symptoms over time, how do you feel?**

Mark only one oval.

- This would be very helpful to me
- This is a basic requirement for me
- This would not affect me
- This would be a minor inconvenience
- This would be a major problem for me

15. **If the app COULD NOT allow you to track your pain symptoms over time, how do you feel?**

Mark only one oval.

- This would be very helpful to me
- This is a basic requirement for me
- This would not affect me
- This would be a minor inconvenience
- This would be a major problem for me

16. **How important is it for the app to allow you to track your pain symptoms over time?**

Mark only one oval.

	1	2	3	4	5	6	7	8	9	
Not at all important										Extremely important

17. **Comments**

5)

18. **If the app could allow you to track your stiffness symptoms over time, how do you feel?**

Mark only one oval.

- This would be very helpful to me
- This is a basic requirement for me
- This would not affect me
- This would be a minor inconvenience
- This would be a major problem for me

19. **If the app COULD NOT allow you to track your stiffness symptoms over time, how do you feel?**

Mark only one oval.

- This would be very helpful to me
- This is a basic requirement for me
- This would not affect me
- This would be a minor inconvenience
- This would be a major problem for me

20. **How important is it for the app to allow you to track your stiffness symptoms over time?**

Mark only one oval.

	1	2	3	4	5	6	7	8	9	
Not at all important	⬭	⬭	⬭	⬭	⬭	⬭	⬭	⬭	⬭	Extremely important

21. **Comments**

6)

22. **If the app could allow you to track your functional impairment symptoms over time, how do you feel?**

Mark only one oval.

- This would be very helpful to me
- This is a basic requirement for me
- This would not affect me
- This would be a minor inconvenience
- This would be a major problem for me

23. **If the app COULD NOT allow you to track your functional impairment symptoms over time, how do you feel?**

Mark only one oval.

- This would be very helpful to me
- This is a basic requirement for me
- This would not affect me
- This would be a minor inconvenience
- This would be a major problem for me

24. **How important is it for the app to allow you to track your functional impairment symptoms over time?**

Mark only one oval.

	1	2	3	4	5	6	7	8	9	
Not at all important	⬭	⬭	⬭	⬭	⬭	⬭	⬭	⬭	⬭	Extremely important

25. **Comments**

7)

26. **If the app could show you a graph of your symptoms over time, how do you feel?**
Mark only one oval.

- This would be very helpful to me
- This is a basic requirement for me
- This would not affect me
- This would be a minor inconvenience
- This would be a major problem for me

27. **If the app COULD NOT show you a graph of your symptoms over time, how do you feel?**
Mark only one oval.

- This would be very helpful to me
- This is a basic requirement for me
- This would not affect me
- This would be a minor inconvenience
- This would be a major problem for me

28. **How important is it for the app to show you a graph of your symptoms over time?**
Mark only one oval.

	1	2	3	4	5	6	7	8	9	
Not at all important										Extremely important

29. **Comments**

8)

30. **If the app could give you strategies to help you self-manage your arthritis, how do you feel?**

Mark only one oval.

- This would be very helpful to me
- This is a basic requirement for me
- This would not affect me
- This would be a minor inconvenience
- This would be a major problem for me

31. **If the app COULD NOT give you strategies to help you self-manage your arthritis, how do you feel?**

Mark only one oval.

- This would be very helpful to me
- This is a basic requirement for me
- This would not affect me
- This would be a minor inconvenience
- This would be a major problem for me

32. **How important is it for the app to give you strategies to help you self-manage your arthritis?**

Mark only one oval.

	1	2	3	4	5	6	7	8	9	
Not at all important										Extremely important

33. **Comments**

9)

34. **If the app could let you 'flag' certain days where arthritis impacted your plans, how do you feel?**

Mark only one oval.

- This would be very helpful to me
- This is a basic requirement for me
- This would not affect me
- This would be a minor inconvenience
- This would be a major problem for me

35. **If the app COULD NOT let you 'flag' certain days where arthritis impacted your plans, how do you feel?**

Mark only one oval.

- This would be very helpful to me
- This is a basic requirement for me
- This would not affect me
- This would be a minor inconvenience
- This would be a major problem for me

36. **How important is it for the app to let you 'flag' certain days where arthritis impacted your plans?**

Mark only one oval.

	1	2	3	4	5	6	7	8	9	
Not at all important										Extremely important

37. **Comments**

10)

38. **If the app could give you reminders to update your information (symptoms, exercise, goal tracking), how do you feel?**

Mark only one oval.

- This would be very helpful to me
- This is a basic requirement for me
- This would not affect me
- This would be a minor inconvenience
- This would be a major problem for me

39. **If the app COULD NOT give you reminders to update your information (symptoms, exercise, goal tracking), how do you feel?**

Mark only one oval.

- This would be very helpful to me
- This is a basic requirement for me
- This would not affect me
- This would be a minor inconvenience
- This would be a major problem for me

40. **How important is it for the app to give you reminders to update your information (symptoms, exercise, goal tracking)?**

Mark only one oval.

	1	2	3	4	5	6	7	8	9	
Not at all important										Extremely important

41. **Comments**

Powered by
Google Forms
