# Supplementary material for: Co-Design in the Development of a Mobile Health App for the Management of Knee Osteoarthritis by Patients and Physicians: Qualitative Study
Source: JMIR Mhealth Uhealth. 2020 Jul 10;8(7):e17893. doi: 10.2196/17893 (PMC7382016; doi:10.2196/17893)
Supplement: Multimedia Appendix 2 [file mhealth_v8i7e17893_app2.pdf]

Table 6 Kano survey findings: Summary of *convenience* response frequencies presession and postsession 3 by functional requirement

	App Feature	Cat	Patients (n=6, 7)		Physicians (n=3, 2)		Researchers (n=3, 3)		Overall (n=13, 12)		Rank[a]
			Pre	Post	Pre	Post	Pre	Post	Pre	Post	
1	If the app could show you a graph of your 7-year osteoarthritis severity prediction, how do you feel?	MH<br>P<br>A<br>I<br>R<br>Q	0<br>1<br>5<br>0<br>0<br>0	0<br>0<br>5<br>2<br>0<br>0	0<br>0<br>1<br>2<br>0<br>0	0<br>0<br>1<br>1<br>0<br>0	0<br>0<br>1<br>2<br>0<br>0	1<br>0<br>1<br>1<br>0<br>0	0<br>1<br>7<br>6<br>0<br>0	1<br>0<br>7<br>4<br>0<br>0	8
2	If the app could help you to set goals and follow through, how do you feel?	MH<br>P<br>A<br>I<br>R<br>Q	0<br>3<br>2<br>1<br>0<br>0	1<br>3<br>1<br>2<br>0<br>0	1<br>1<br>1<br>0<br>0<br>0	0<br>1<br>1<br>0<br>0<br>0	1<br>0<br>2<br>0<br>0<br>0	1<br>0<br>0<br>2<br>0<br>0	2<br>4<br>5<br>1<br>0<br>0	2<br>4<br>2<br>4<br>0<br>0	5
3	If the app could help you set a plan with various exercises and track them daily, how do you feel?	MH<br>P<br>A<br>I<br>R<br>Q	1<br>2<br>1<br>2<br>0<br>0	1<br>2<br>1<br>2<br>0<br>1	1<br>2<br>0<br>0<br>0<br>0	0<br>2<br>0<br>0<br>0<br>0	1<br>0<br>0<br>2<br>0<br>0	2<br>0<br>0<br>1<br>0<br>0	3<br>4<br>1<br>4<br>0<br>0	3<br>4<br>1<br>3<br>0<br>0	3
4	If the app could allow you to track your pain symptoms over time, how do you feel?	MH<br>P<br>A<br>I<br>R<br>Q	0<br>2<br>2<br>2<br>0<br>0	3<br>1<br>1<br>2<br>0<br>0	0<br>1<br>1<br>1<br>0<br>0	0<br>1<br>1<br>0<br>0<br>0	1<br>2<br>0<br>0<br>0<br>0	1<br>1<br>0<br>0<br>0<br>1	1<br>5<br>3<br>3<br>0<br>0	4<br>3<br>2<br>2<br>0<br>1	2
5	If the app could allow you to track your stiffness symptoms over time, how do you feel?	MH<br>P<br>A<br>I<br>R<br>Q	0<br>1<br>3<br>2<br>0<br>0	0<br>0<br>4<br>3<br>0<br>0	0<br>0<br>0<br>3<br>0<br>0	0<br>0<br>0<br>2<br>0<br>0	0<br>0<br>2<br>1<br>0<br>0	1<br>1<br>1<br>0<br>0<br>0	0<br>1<br>5<br>6<br>0<br>0	1<br>1<br>5<br>5<br>0<br>0	9

6	If the app could allow you to track your functional impairment symptoms over time, how do you feel?	MH<br>P<br>A<br>I<br>R<br>Q	0<br>3<br>1<br>2<br>0<br>0	0<br>1<br>3<br>3<br>0<br>0	1<br>1<br>0<br>1<br>0<br>0	0<br>2<br>0<br>0<br>0<br>0	1<br>1<br>0<br>1<br>0<br>0	2<br>1<br>0<br>0<br>0<br>0	2<br>5<br>1<br>4<br>0<br>0	2<br>4<br>3<br>3<br>0<br>0	6
7	If the app could show you a graph of your symptoms over time, how do you feel?	MH<br>P<br>A<br>I<br>R<br>Q	0<br>3<br>2<br>1<br>0<br>0	2<br>2<br>1<br>2<br>0<br>0	1<br>0<br>1<br>1<br>0<br>0	1<br>0<br>0<br>1<br>0<br>0	0<br>2<br>0<br>1<br>0<br>0	1<br>1<br>2<br>1<br>0<br>0	1<br>5<br>3<br>3<br>0<br>0	5<br>2<br>2<br>3<br>0<br>0	1
8	If the app could give you strategies to help you self-manage your arthritis, how do you feel?	MH<br>P<br>A<br>I<br>R<br>Q	0<br>2<br>2<br>2<br>0<br>0	1<br>1<br>3<br>2<br>0<br>0	0<br>2<br>1<br>0<br>0<br>0	0<br>0<br>2<br>0<br>0<br>0	0<br>1<br>2<br>0<br>0<br>0	0<br>1<br>0<br>2<br>0<br>0	0<br>5<br>5<br>2<br>0<br>0	2<br>1<br>9<br>3<br>0<br>0	7
9	If the app could let you *flag* certain days where arthritis impacted your plans, how do you feel?	MH<br>P<br>A<br>I<br>R<br>Q	0<br>0<br>1<br>5<br>0<br>0	0<br>0<br>2<br>5<br>0<br>0	0<br>0<br>2<br>1<br>0<br>0	0<br>0<br>0<br>2<br>0<br>0	0<br>2<br>0<br>1<br>0<br>0	0<br>1<br>0<br>2<br>0<br>0	0<br>2<br>3<br>7<br>0<br>0	0<br>1<br>2<br>9<br>0<br>0	10
10	If the app could give you reminders to update your information (symptoms, exercise, goal tracking), how do you feel?	MH<br>P<br>A<br>I<br>R<br>Q	1<br>1<br>2<br>2<br>0<br>0	1<br>0<br>1<br>5<br>0<br>0	0<br>1<br>1<br>1<br>0<br>0	1<br>0<br>0<br>1<br>0<br>0	0<br>0<br>2<br>1<br>0<br>0	0<br>0<br>1<br>1<br>0<br>1	1<br>2<br>5<br>4<br>0<br>0	2<br>0<br>2<br>7<br>0<br>1	4

Cat (Category): MH = Must Have, P = Performance, A = Attractive, I = Indifferent, R = Reverse, Q = Questionable

[a]Rank subjectively determined by the combination of MH, P, A feature prioritizations for each functional requirement, and whether the features increased, stayed the same or decreased in frequency between pre- and post-session surveys
