# Supplementary figures and images for: Co-Design in the Development of a Mobile Health App for the Management of Knee Osteoarthritis by Patients and Physicians: Qualitative Study
Source: JMIR Mhealth Uhealth. 2020 Jul 10;8(7):e17893. doi: 10.2196/17893 (PMC7382016; doi:10.2196/17893)

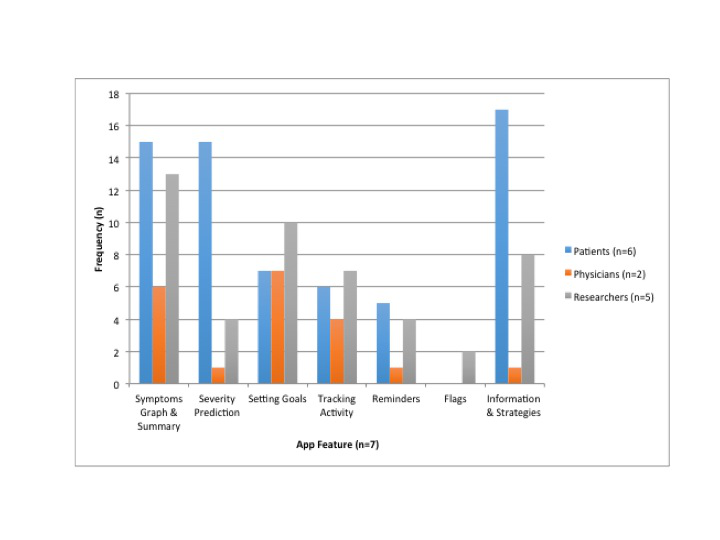

Supplement: Multimedia Appendix 3 [file mhealth_v8i7e17893_app3.png]

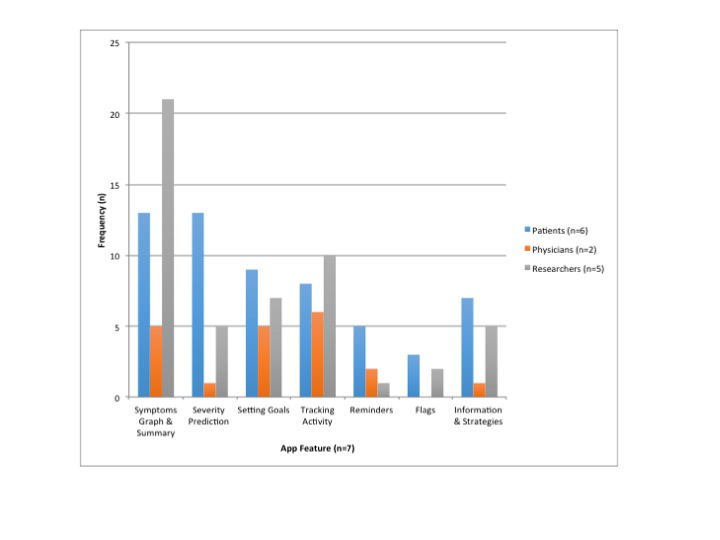

Supplement: Multimedia Appendix 4 [file mhealth_v8i7e17893_app4.png]
